# The Bernese Motive and Goal Inventory for Adolescence and Young Adulthood

**DOI:** 10.3389/fpsyg.2018.02785

**Published:** 2019-01-24

**Authors:** Vanessa Gut, Julia Schmid, Jürg Schmid, Achim Conzelmann

**Affiliations:** Institute of Sport Science, University of Bern, Bern, Switzerland

**Keywords:** sport- and exercise-related motives and goals, German questionnaire, physical activity, motivation, exploratory structural equation modeling, adolescence, early adulthood, measurement invariance

## Abstract

Exercise- and sport-related motives and goals are important motivational factors in promoting exercise and sport among adolescents and young adults. However, at present, there is no well-validated instrument to assess these factors that considers age-specific characteristics. Therefore, the goals of this study were to adapt the existing Bernese Motive and Goal Inventory in exercise and sport for middle-aged adults for use in adolescents and young adults and to examine its psychometric properties. The Bernese Motive and Goal Inventory for adolescence and young adulthood was validated with 2,318 participants aged between 14 to 34 years old. Applying exploratory structural equation modeling, the inventory demonstrated excellent model fit (CFI = 0.983, SRMR = 0.014, RMSEA = 0.040) using 26 items and covering eight motives and goals: Contact, Competition/Performance, Distraction/Catharsis, Body/Appearance, Health, Fitness, Aesthetics, and Risk/Challenge. A cross-validation confirmed the factor structure. Psychometric analyses revealed good reliabilities (CR ≥ 0.70, AVE ≥ 0.50, test-retest reliability: 0.62 ≤ *r*_tt_ ≤ 0.83) and discriminant validity. The factors correlated, in predictable ways, with exercise- and sport-related self-concordance, indicating criterion validity of the inventory. Additionally, metric measurement invariance was supported for activity levels, gender, and age. Overall, the Bernese Motive and Goal Inventory for adolescence and young adulthood is an age-specific, economical, and psychometrically sound questionnaire to assess exercise- and sport-related motives and goals. The inventory can be used in the practical field of exercise and sport promotion (e.g., sport counseling), as well as in research, to better understand the mechanisms and effects of motives and goals in exercise and sport.

## Introduction

Focusing on adolescents and young adults when promoting exercise and sport seems to be especially meaningful in view of the various positive long-term effects on psychosocial and physical health (e.g., Lubans et al., 2016; Warburton and Bredin, 2017). From a public health perspective, it is therefore important to understand the forces that drive adolescents and young adults to become and to remain physically active.

Motives and goals play an important role for adolescents and young adults in driving exercise and sport behavior. Explicit motives can be defined as self-attributed needs and conscious goals, which may be classified as a middle level in a hierarchy of goals (Heckhausen and Heckhausen, 2008). Goals are “internal representations of desired states, where states are broadly construed as outcomes, events, or processes” (Austin and Vancouver, 1996; p. 338). The field of exercise and sport provides many opportunities to satisfy a wide range of individual motives and to attain a great diversity of goals, such as competition, distraction, health promotion, or fitness improvement (e.g., Lehnert et al., 2011). Several studies have shown that satisfying exercise- and sport-related motives and attaining goals positively influence affective well-being (Sudeck and Conzelmann, 2011; Gunnell et al., 2014; Antunes et al., 2018). Improved affective well-being, in turn, has a positive effect on exercise and sport adherence (Rhodes and Kates, 2015). Therefore, practitioners in the field of exercise and sport promotion (such as sport counselors), as well as researchers who want to better understand the central role of motives and goals, need tools that will enable them to assess a broad variety of exercise- and sport-related motives and goals.

To our knowledge, no well-validated age-specific questionnaire exists that can readily be used for German-speaking clients and populations. There are several instruments in the published literature, as detailed in the Electronical Supplementary Material (ESM) 1. However, all these questionnaires have at least one or more shortcomings. First, some questionnaires lack a theoretical background (e.g., Gill et al., 1983). Second, some questionnaires have a rather low test efficiency (e.g., Schmid et al., 2017), which might lead to acceptance problems in the field of practice as well as research. Third, several questionnaires show critical test-statistical criteria, such as low internal consistency (e.g., Gill et al., 1983; Steffgen et al., 2000; Ingledew and Sullivan, 2002) or restricted factorial validity (e.g., Kueh et al., 2018). Fourth, questionnaires have rarely been tested for measurement invariance, although it is a precondition to compare motives and goals across different groups (Marsh et al., 2009). Fifth, age-specific motives and goals for adolescents and young adults derived from a developmental psychology perspective have not been considered within questionnaire development (e.g., Kueh et al., 2018). Despite these limitations, it is preferable to build on existing preliminary work than to start completely from the beginning.

We considered the Bernese Motive and Goal Inventory in exercise and sport for middle adulthood, that is, individuals aged between 35 to 64 years (Berner Motiv-und Zielinventar [BMZI]: Lehnert et al., 2011) as the most promising option to develop a German instrument, for two reasons: First, the use of a German questionnaire as a foundation reduces potential linguistic and cultural biases. Second, the BMZI is a theoretically-founded, economical and well-validated questionnaire: The inventory is grounded on Gabler's (2002) taxonomy of motives. Gabler identified recurring basic situations in exercise and sport, such as social interaction, which are linked with specific motives and goals. These, in turn, were classified by referring to their instrumental value: motives and goals that may be related to exercise and sport activity itself, to the results of exercise and sport activity, or those that may be seen as a means to further purposes. Thus, the BMZI can be theoretically linked to the distinction between activity-centered vs. purpose-centered incentives (Rheinberg, 2008), and also the distinction between intrinsic vs. extrinsic goal contents (Goal Content Theory as part of the Self-Determination Theory proposed by Ryan and Deci, 2017). The BMZI consists of 24 items covering seven categories of motives and goals, summarized as follows. *Contact*: communicating with friends, meeting new people, and making friends. *Competition/Performance*: comparing oneself to others and improving skills. *Distraction/Catharsis*: distracting oneself from worries and reducing stress. *Body/Appearance:* regulating body weight and shaping the body for a better appearance. *Fitness/Health*: improving fitness and promoting health. *Activation/Enjoyment*: enjoying moving and regaining energy. *Aesthetics*: experiencing beautiful movements, e.g., rhythmic movements during dancing or while skiing. The BMZI was recently updated (Schmid et al., 2018) and has been applied in various settings, such as exercise therapy (Krauss et al., 2017; Schmid et al., 2018) and leisure sport activities (Sudeck and Conzelmann, 2011; Ley and Krenn, 2017; Schmid et al., 2018).

However, based on developmental-psychological considerations (Heckhausen et al., 2010; Newman and Newman, 2012; Shaffer and Kipp, 2014; Arnett, 2016;) and empirical findings (Trujillo et al., 2004; Quindry et al., 2011; Stults-Kolehmainen et al., 2013; Molanorouzi et al., 2015), it seems clear that the motives and goals from middle adulthood cannot be uncritically transferred to adolescence and young adulthood. The developmental tasks of adolescents and young adults (e.g., exploring identity, conducting intimate relationships, finding one's own lifestyle) are very different to the tasks of middle adulthood (e.g., bringing up children, pursuing a professional career) (Havighurst, 1972). Individuals at different life stages have different life goals and find themselves in different contexts (Krings et al., 2008). Thus, the relevance of different exercise- and sport-related motives and goals varies correspondingly.

As a result, the age-specific adaptation of the BMZI to focus is on adolescents from the age of 14 years to young adults, that is, those aged up to 34 years, is necessary (Newman and Newman, 2012). This adaptation is guided by three assumptions: First, some motives and goals have greater significance, whereas other motives and goals tend to be more marginal in adolescence and young adulthood (Campbell et al., 2001; Lehnert et al., 2011). Second, some motives and goals may be cognitively more differentiated (Lehnert et al., 2011). Third, additional and other age-specific motives and goals for adolescents and young adults may also be relevant.

The overarching goals of this study were to adapt the BMZI for adolescents and young adults aged 14–34 years and to examine its psychometric properties. In particular, six aims were pursued (see Table 1 for an overview). First, for the adaptation of the BMZI it was necessary to identify age-specific motives and goals and to develop an initial item pool. Second, the factorial structure of the instrument was analyzed. The initial items were explored; then, after some unsatisfactory items were revised or exchanged against new ones, the factorial structure was again explored and cross-validated. Third, different types of reliabilities were estimated. Fourth, the discriminant validity of the BMZI for adolescence and young adulthood was investigated. Fifth, criterion validity was examined by investigating the relationship between motives and goals and also the construct of exercise- and sport-related self-concordance. The latter may be defined as the degree of closeness of a chosen goal with personal interests and values (Sheldon and Elliot, 1999). Following this definition, self-concordance covers four different modes of motivation, which are placed on a continuum from low to high self-concordant goals. In the intrinsic mode, the incentive is inherent in the goal itself. In the identified mode, a goal is completely integrated into personal interests and values. In contrast, in the introjected mode goals are chosen because they are considered meaningful but do not represent personal interests and values. In the extrinsic mode, goals are pursued only because of external incentives. Based on this theoretical consideration as well as on empirical findings (Lehnert et al., 2011; Schmid et al., 2014, 2018), the following hypotheses were formulated: (a) activity-centered motives (e.g., Competition/Performance, Aesthetics) are predominantly related to the intrinsic mode because the incentive is inherent in the activity; (b) motives and goals which are meaningful for personal values and interests (e.g., Health/Fitness) are related to the introjected or identified mode; and (c) purpose-centered motives and goals (e.g., Body/Appearance) are associated with the extrinsic mode. Sixth, the measurement invariance across activity levels, gender, and age was tested.

**Table 1:** Aims of the study.

**Overarching goals**	**Adaptation of the BMZI**	**Examination of psychometric properties**				
Aims	1. Identification of relevant topics and development of an initial item pool	2. Factorial validity	3. Reliabilities	4. Discriminant validity	5. Criterion validity	6. Measurement invariance
Procedure	(a) Identification of need for age-specific adaptation of the BMZI (b) Test of comprehensibility	Examination of: (a) Initial factorial validity (b) Factorial validity revised with an adapted item pool (c) Cross-validation	Examination of: (a) Reliability of the factors (b) Reliability of the indicators (c) Test-retest reliability	Examination of discriminant validity	Examination of criterion validity (validation with sport- and exercise-related self-concordance)	Examination of measurement invariance across activity levels, gender, and age
Method	(a) Expert focus group and screening of relevant literature (b) Think-aloud-interviews to test the comprehensibility of the items among adolescents	(a,b) ESEM with geomin rotation (c) *R*_V_ coefficient for cross-validation	(a) Composite reliability, average variance explained (b) Squared multiple correlations (c) Correlation of factor scores	Fornell-Larcker criterion, HTMT ratio	Correlations of factor scores	ESEM with geomin rotation
Sample	(a) 8 sport scientists and 4 psychologists (b) 4 adolescents	(a) Sample A (*n* = 700) (b) Sample B (*n* = 788) (c) Sample C (*n* = 830)	(a,b) Sample B (*n* = 788) (c) Subsample C (*n* = 265)	Sample B (*n* = 788)	Sample B (*n* = 788)	Samples B (*n* = 788) and C (*n* = 830)

*BMZI, Bernese Motive and Goal Inventory; ESEM, exploratory structural equation modeling; HTMT ratio, heterotrait-monotrait ratio*.

## Methods

### Initial Item Development

To identify potentially important motives and goals for adolescents and young adults, the following approach was used: First, a focus group (Morgan and Krueger, 1998; Barbour, 2007) with eight sport scientists and four psychologists was conducted. Second, a review of the relevant literature in the field of developmental psychology was conducted. The results of both procedures were compared and discussed by the authors. Finally, the following needs for the adaptation of the original BMZI were identified.

#### Differentiation in Fitness and Health

Adolescents and young adults may have an understanding of health that has rather negative connotations, whereby the pathogenesis of diseases is the focus. In addition, fitness has a more positive connotation and is associated with an active lifestyle (Michaud et al., 2006; Ott et al., 2011). As a result, we pursued a differentiation in Fitness and Health.

#### Differentiation in Competition and Performance

Achievement and individual performance enhancement is a central topic across the whole of a person's life (Steinberg et al., 2001). However, to compete with other peers and to be better than others are especially important topics for adolescents and young adults (Steinberg, 2016), in particular with regard to the field of exercise and sport (Weiss and Williams, 2004; Quindry et al., 2011; Molanorouzi et al., 2015). Therefore, based on the importance of competition in this stage of life, we pursued a differentiation in Competition and Performance.

#### Differentiation in Body Weight and Appearance

In adolescence and young adulthood, a growing importance of body image may be observed, caused by physical changes and by comparisons to the body ideals of society (Jackson and Goossens, 2006; Ricciardelli and Yager, 2016). As a result, adolescents and young adults increasingly want to regulate their body weight and improve their appearance. Therefore, we assumed that adolescents and young adults have a more differentiated cognitive representation of this motive and goal (Lehnert et al., 2011) that may result in a differentiation in Body Weight and Appearance.

#### Addition of the New Motive and Goal, Risk/Challenge

Adolescents and young adults often look for risky situations and tend to engage in more experimental and exploratory behavior than older adults (Michaud et al., 2006; Rodham et al., 2006; Pharo et al., 2011; Arnett, 2016). Exercise and sport activities, in particular, provide challenging tasks for young people to live out such risk-taking behavior. Thus, we intended to form a new facet called Risk/Challenge.

Based on the identified topics, a pool of potential items was generated. This led to a total of 41 items: 17 new items and 24 items from the original BMZI (see Appendix). These items were validated communicatively with four adolescents in terms of comprehensibility using the think-aloud technique (Presser et al., 2004). As a result, one item of the original BMZI was excluded due to its linguistic complexity (item: “[I exercise/do sport] for the enjoyment of beautiful movements in exercise and sport”). In accordance with the BMZI, participants were asked: “Why do you exercise or do sport/Why would you exercise or do sport?” and invited to indicated the extent to which they agreed with the remaining 40 statements (for example, “To do something in a group”) on a Likert scale ranging from 1 (“*I strongly disagree”*) to 5 (“*I strongly agree”*).

### Participants and Procedure

To examine the psychometric properties of the questionnaire, three different samples (A, B, C) and a subsample of C were recruited in the German-speaking part of Switzerland. A detailed description of all samples is presented in Table 2. In line with the targeted age range, participants younger than 14 years and older than 34 years were excluded. Furthermore, participants were excluded if they had physical disabilities that prevented them from exercising or participating in sport on a regular basis, or if they did not have basic skills in German. The students and employees among the participants were recruited via personal contact with teachers of public schools as well as vocational education and training, university lecturers, and human resources managers from agencies and services companies. Students at public schools and universities were invited at a class or lecture, respectively, to complete a paper-pencil version of the questionnaire supervised by a trained researcher. In contrast, individuals of the university of applied science, service companies, and administration agencies were personally contacted by email or health management platforms to fill in an online-version of the questionnaire. For practical and organizational reasons, it was not possible to use uniform instrumentation for all participants. All participants gave their written informed consent and were free to decline participation. Additionally, all adolescents below 16 years of age were required to obtain written informed consent from their parents to participate. The Ethics Committee of the University of Bern's Faculty of Human Sciences approved the study.

**Table 2:** Characteristics of participants and procedures.

	**Sample A**	**Sample B**	**Sample C**	**Subsample C**
Sample size	*n* = 727	*n* = 826	*n* = 886	*n* = 274
Gender	59% female; 41% male	60% female; 40% male	57% female; 43% male	58% female; 42% male
Age (*M, SD*, range)	*M* = 20.56 years; *SD* = 5.87; range = 14–34 years	*M* = 19.55 years; *SD* = 4.32; range = 14–34 years	*M* = 19.57 years; *SD* = 4.94; range = 14–34 years	*M* = 18.02 years; *SD* = 2.82; range = 15–32 years
Percentage of physically inactive individuals^a^	22%	24%	26%	23%
Data collection period	9/2015–2/2016	3/2016–8/2017	9/2016–1/2017	2/2017–6/2017
Type and sources of data collection	Paper-pencil version: 45% public schools (22 classes) 15% one university ]Online version: 12% university of applied science 17% two service companies 11% public administration agency	Paper-pencil version: 48% public schools (23 classes) 31% one university Online version: 6% vocational school 9% university of applied science 7% two service companies	Paper-pencil version: 27% public schools (15 classes) 6% one university ]Online version: 12% university of applied science 60% three service companies 7% public administration agencies	Online version: 7% one university 9% two service companies

*Subsample C was generated by asking 567 adolescents and young adults of Sample C to fill in the inventory a second time after 2 weeks; therefore, 278 individuals participated twice. ^a^Activity level was assessed with the BSA-F from Fuchs et al. (2015)*.

To estimate the sample size needed, the recommendations of Worthington and Whittaker (2006), as well as Tabachnick and Fidell (2013) were considered. They recommend, as a rule of thumb, a sample of 300 for factor analysis or multi-group analysis for the test of measurement invariance (e.g., regarding activity level: physically inactive vs. physically active), respectively. As approximately 20% of the Swiss population are physically inactive (Lamprecht et al., 2014), at least 1,500 individuals should be recruited to obtain a sample of 300 inactive adolescents and young adults, and thus reliable results.

### Measures

To validate the BMZI for adolescence and young adulthood with an external criterion, the self-concordance of exercise- and sport-related goals was assessed using a well-validated German questionnaire (Seelig and Fuchs, 2006). Comprising 12 items and four subscales, it measures four modes of motivation for exercise and sport: intrinsic, identified, introjected, and external (73 ≤ α ≤ 0.81, calculation based on sample B). The participants ranked their self-concordance on a Likert scale from 1 (“*I strongly disagree”*) to 6 (“*I strongly agree”*).

To examine measurement invariance of the BMZI in regard to activity level, the Physical Activity, Exercise, and Sport Questionnaire (Bewegungs- und Sportaktivitäts-Fragebogen; BSA-F) from Fuchs et al. (2015) was used to assess exercise and sport activities. The participants were asked to record a maximum of three activities and to indicate how many times in the 4 weeks prior to data collection they engaged in each exercise or sport activity, and for how many minutes.

### Data Analyses

#### Factorial Validity

To examine the initial factor structure of the item pool, exploratory structural equation modeling (ESEM) (Asparouhov and Muthén, 2009) was applied with sample A using Mplus 8 (Muthén and Muthén, 1998–2018). ESEM is a relatively new statistical analysis for questionnaire development that integrates the advantages of exploratory and confirmatory factor analysis (CFA) (Marsh et al., 2014; Myers et al., 2017). ESEM allows cross-loadings; thus, it represents the underlying structure more realistically than CFA and provides a better model fit (Marsh et al., 2009). It is especially recommended for multidimensional instruments with correlated factors (Marsh et al., 2014). An oblique geomin rotation (ε = 0.5) with robust maximum likelihood estimation for all ESEM analyses was used. To test model fit, three measures were used: the comparative fit index (CFI); root mean square error of approximation (RMSEA); and standardized root mean square residual (SRMR). Following Schermelleh-Engel et al.'s (2003) recommendations for model evaluation, as indicators of good fit the cut-off criteria of >0.97 for CFI, <0.05 for RMSEA, and <0.05 for SRMR were chosen to indicate a good fit. On a more detailed level, items with standardized factor loadings <0.50 and cross-loadings >0.30 were excluded. For more specific information concerning the comparison of different competing models, see the ESM 2.

After revising the item pool (see Initial Item Development), the factorial structure of the inventory was again analyzed (sample B) and cross-validated (sample C) using ESEM. The similarity of the two-factor loading matrices was evaluated using the *R*_*V*_ coefficient from the R package FactoMineR version 1.36 (Husson et al., 2018).

#### Reliabilities

To examine the reliability of the factors, the composite reliability (CR: Bagozzi and Yi, 2012) and the average variance explained (AVE: Fornell and Larcker, 1981) was calculated. To examine the reliability of the indicators, squared multiple correlations (SMC) were estimated. The following cut-offs for good reliabilities were used: CR ≥ 0.70 (Bagozzi and Yi, 2012); AVE ≥ 0.50 (Bagozzi and Yi, 2012); and SMC ≥ 0.50 (Hair et al., 2010). While theses analyses were based on sample B, Subsample C was used to examine the test-retest reliability over a 2-weeks period using Pearson correlation coefficients.

#### Discriminant Validity

To check for discriminant validity, the Fornell–Larcker criterion was used and it was examined whether the AVE of each factor was greater than the squared variance of all the other factors (Fornell and Larcker, 1981). Furthermore, the heterotrait-monotrait (HTMT) ratio (Henseler et al., 2015) with a cut-off value of 0.85 (Kline, 2011) was applied. The HTMT ratio “is the average of the heterotrait-heteromethod correlations … relative to the average of the monotrait-heteromethod correlations” (Henseler et al., 2015). All these analyses were based on sample B.

#### Criterion Validity

To investigate the criterion validity, correlation coefficients (Pearson's *r*) between the eight factor scores representing the BMZI for adolescence and young adulthood and the four factor scores measuring the modes self-concordance were calculated (sample B).

#### Measurement Invariance

As a precondition, measurement invariance across samples B and C was examined. If the measurement invariance across the two samples may be assumed, they will be merged into one large sample for the actual analyses of the measurement invariance. The aim was to apply the questionnaire to active and inactive as well as male and female individuals in the age range 14–34 years. Therefore, the sample was split for each of the three variables into two groups and tested the measurement invariance across the groups as follows: a physically inactive group (no time spent on any exercise and sport activities) vs. an active group (any amount of time spent on exercise and sport); females vs. males; and a younger group (14–19 years) vs. an older group (20–34 years). Following (Marsh et al., 2009) taxonomy of multiple group tests of invariance testable with ESEM, configural, metric, and scalar measurement invariance was examined using simultaneous estimations of models. Measurement invariance across two samples can be assumed if the difference of the fit indices is ≤ 0.010 for CFI and ≤ 0.015 for RMSEA, respectively (Chen, 2007).

#### Data Preparation

All samples were checked for multivariate outliers using Mahalanobis distance values as χ^2^ at *p* < 0.001 (Tabachnick and Fidell, 2013). This criterion led to the exclusion of 27 individuals in sample A, 38 individuals in sample B, 56 individuals in sample C, and 9 individuals in subsample C. Missing values were <5% and were estimated using the full information maximum likelihood procedure (Little and Rubin, 2012).

## Results and Brief Discussion

### Factorial Validity

The analyses of the initial factorial validity showed that ESEM yielded a good fit (CFI = 0.983, SRMR = 0.016, RMSEA = 0.042, 90% CI [035–0.049]) for a model with seven factors consisting of 25 items (ESM 3). The seven factors were: Contact; Competition/Performance; Distraction/Catharsis; Body/Appearance; Health; Fitness; and Aesthetics.

A thorough inspection of this factor solution revealed several particularities. The three items under Activation/Enjoyment had several cross-loadings and did not clearly define the intended factor. This finding is in line with Lehnert et al. (2011) which found the factor Activation/Enjoyment to have low factorial, convergent, and divergent validity and, as a consequence, called for a critical reappraisal of this factor. Therefore, the three items concerning Activation/Enjoyment (see Appendix) were excluded. A further peculiarity was that the factors Body/Weight and Appearance could not be differentiated easily empirically (see ESM 2). In the absence of better items, the original ones from the Body/Appearance factor of the BMZI were retained. Conversely, an empirical differentiation in Health and Fitness, as well as a differentiation trend in Competition and Performance, became apparent (see ESM 2). Finally, the factor Risk/Challenge was recognizable but not clearly distinguishable from the factor Competition/Performance (see ESM 2). Thus, the focus group of expert sport scientists and psychologists generated three additional items that were thought to more clearly measure the motives and goals of Risk/Challenge (two items) as opposed to Competition and Performance (one item; see Appendix).

After the exclusion of items in the check for factorial validity among sample B, the final ESEM model consisted of eight motives and goals, comprising 26 items in total (see Table 3): Contact, Competition/Performance, Distraction/Catharsis, Body/Appearance, Health, Fitness, Aesthetics, and Risk/Challenge. Despite the added item “To increase my level of performance” (comper7), no differentiation of Competition and Performance was found. The fit of the data to the eight-factor solution was good (CFI = 0.983, SRMR = 0.014, RMSEA = 0.040, 90% CI [0.034–0.045]); for more information concerning alternative factor solutions, see ESM 2. With respect to their standardized factor loadings (ranging from 0.55 to 0.91), all items exceeded the cut-off value of 0.50. Nevertheless, there was one exception: the item “To achieve my exercise goals” (comper3, λ = 0.45), which was also characterized by a substantial cross-loading on the factor Fitness (λ = 0.38). Furthermore, the factorial structure of sample B was essentially equal to that of sample C (*R*_V_ coefficient = 0.97; *p* < 0.001).

**Table 3:** Standardized factor loadings of sample B (*n* = 788) using exploratory structural equation modeling.

**Items**	**Factors**								**SMC**
	**Contact**	**Competition/performance**	**Distraction/catharsis**	**Body/appearance**	**Health**	**Fitness**	**Aesthetics**	**Risk/challenge**	
con1	0.78								0.70
con2	0.80								0.78
con3	0.82								0.73
con4^a^	0.55								0.46
con5^a^	0.55								0.48
comper1		0.87							0.79
comper2		0.65							0.58
comper3		0.45				0.38			0.49
discat1			0.68						0.63
discat2			0.68						0.82
discat3			0.65						0.52
discat4			0.70						0.53
bodapp1				0.87					0.75
bodapp2				0.87					0.85
bodapp3				0.65		0.20			0.66
hea1					0.81				0.80
hea2					0.75				0.70
hea3					0.59				0.44
fit1						0.67			0.57
fit2						0.76			0.73
fit3						0.71			0.75
aes1							0.91		0.87
aes2							0.77		0.60
rischa1								0.66	0.60
rischa2								0.71	0.65
rischa3								0.86	0.82

*SMC, squared multiple correlations; loadings < 0.20 are not presented. ^a^In accordance with the studies of the BMZI for middle adulthood (Lehnert et al., 2011; Schmid et al., 2018) and older adulthood (Schmid et al., 2014), an error covariance between con4 and con5 was permitted*.

The findings indicate a clear factorial structure of the BMZI for adolescence and young adulthood, with eight motives and goals: Contact, Competition/Performance, Distraction/Catharsis, Body/Appearance, Health, Fitness, Aesthetics, and Risk/Challenge. Concerning the standardized factor loadings, only one item had a noticeable cross-loading on Fitness (comper3). For reasons of comparability of the factor Competition/Performance across middle and late adulthood (Lehnert et al., 2011; Schmid et al., 2014, respectively), the item was retained.

### Reliabilities

The examination of the reliabilities demonstrated good to very good values for all eight factors (see Table 4; sample B). All the values of CR and AVE met the cut-off criteria, ranging from 0.77 to 0.89 (ρ) for CR and from 0.56 to 0.84 (ρ) for AVE, respectively. The indicator reliabilities were good, except for four items (SMC_con4_ = 0.46, SMC_con5_ = 0.48, SMC_comper3_ = 0.49, and SMC_hea3_ = 0.44), which were slightly lower than recommended (see Table 3). The test-retest reliability (*r*_tt_) of the eight subscales of subsample C over a 2-weeks period showed correlations, ranging from 0.62 to 0.83 (see Table 4). Although the three subscales Fitness (*r*_tt_ = 0.62), Aesthetics (*r*_tt_ = 0.69), and Risk/Challenge (*r*_tt_ = 0.69) fell short of the recommended cut-off value of 0.70, the results indicate overall a satisfactory test-retest reliability of the BMZI for adolescence and young adulthood.

**Table 4:** Descriptive statistics, reliabilities, correlations of scales, and correlations of factor scores between modes of motivation and motives/goals of the BMZI for adolescence and young adulthood.

**Motives and goals (number of items)**	**Descriptive statistics**		**Reliabilities**			**Correlations of scales (*****r*****)**							**Correlations of factor scores (*****r*****)**			
						**Motives and goals**							**Modes of motivation**			
	***M***	***SD***	**CR (ρ)**	**AVE (ρ)**	***r*_**tt**_**	**2**	**3**	**4**	**5**	**6**	**7**	**8**	**Intrinsic**	**Identified**	**Introjected**	**Extrinsic**
1. Contact (5)	2.75	1.07	0.84	0.63	0.77	0.45	0.08	−0.11	−0.04	0.03	0.28	0.36	0.33	0.07	−0.08	0.17
2. Competition/Performance (3)	2.61	1.10	0.77	0.62	0.81	–	0.13	−0.11	−0.02	0.13	0.26	0.51	0.43	0.18	−0.02	0.12
3. Distraction/Catharsis (4)	3.20	1.09	0.86	0.56	0.75		–	0.25	0.31	0.35	0.26	0.24	0.30	0.36	0.26	0.03
4. Body/Appearance (3)	2.92	1.22	0.89	0.75	0.83			–	0.43	0.40	−0.01	−0.08	−0.10	0.26	0.38	0.09
5. Health (3)	3.23	1.07	0.81	0.65	0.72				–	0.58	0.09	0.01	0.03	0.35	0.32	0.13
6. Fitness (3)	4.18	0.82	0.83	0.68	0.62					–	0.14	0.03	0.30	0.57	0.32	−0.03
7. Aesthetics (2)	2.52	1.22	0.88	0.84	0.69						–	0.35	0.36	0.19	0.05	0.19
8. Risk/Challenge (3)	2.10	1.04	0.84	0.69	0.69							–	0.33	0.13	0.07	0.25

*All analyses are based on Sample B (N = 788), except for the test-retest reliability, which used Subsample C (n = 265). CR, composite reliability; AVE, average variance explained; r_tt_, test-retest reliability; 2, Competition/Performance; 3, Distraction/Catharsis; 4, Body/Appearance; 5, Health; 6, Fitness; 7, Aesthetics; 8, Risk/Challenge. Correlations of factor scores show marginal differences compared with correlations of scales (M_differences_ = 0.05, SD_differences_ = 0.05)*.

In the case of the low test-retest reliability of Fitness, an explanation could be that the high mean value and the low standard deviation of Fitness indicates a ceiling effect (*M*_t1_ = 4.18, *SD*_t1_ = 0.78 and *M*_t2_ = 4.00, *SD*_t2_ = 0.86). In the case of Aesthetics and Risk/Challenge, it is possible that these motives and goals are predominately situational. For instance, Jeckel and Sudeck (2017) showed that specific exercise- and sport-related motives and goals may vary across situations within a person.

### Discriminant Validity

All the factors met the Fornell-Larcker criterion (see Table 4, sample B). Concerning the HTMT criterion, six of the eight factors met the cut-off criterion of 0.85, with the ratios ranging from 0.09 to 0.77. The ratios of two factors, Health and Fitness, were 0.88 and slightly higher than recommended. Nevertheless, the discriminant validity of the BMZI for adolescence and young adulthood can be regarded as acceptable.

### Criterion Validity

As expected (hypothesis a), the intrinsic mode of motivation in sample B was linked to activity-centered motives and goals such as Competition/Performance, Aesthetics, and Risk/Challenge (see Table 4). Additionally, the intrinsic mode of motivation was also positively correlated with Contact, Distraction/Catharsis, and Fitness. All factors except for Contact showed small to middle correlations with the identified mode of motivation. The high correlation with Fitness illustrates the strong inherence in the value system of adolescents and young adults. In accordance with Lehnert et al. (2011) and Schmid et al. (2014), medium-sized correlations between Body/Appearance, Health and Fitness, and the introjected mode of motivation were observed (hypothesis b), as well as a small correlation between Distraction/Catharsis and the introjected mode. Contrary to our expectations, no correlation of Body/Appearance with the extrinsic mode of motivation was found (hypothesis c), whereas this mode was positively correlated with Contact, Competition/Performance, Health, Aesthetics, and Risk/Challenge. The differential correlation pattern of Fitness and Health with motivational modes underscores the need to separate the two aspects.

### Measurement Invariance

In the first step, configural measurement invariance was independently demonstrated for samples B and C (see Table 5). The data of the two samples also met the cut-off values of ≤ 0.010 for CFI and ≤ 0.015 for RMSEA, respectively, for the metric and scalar invariance. Based on these findings, the two samples were merged for further tests of invariance. Analogously, metric invariance was demonstrated for all three variables: activity levels, gender, and age. However, the respective scalar model for each of the three variables did meet the cut-off value of ≤ 0.015 for RMSEA, but not the cut-off value of ≤ 0.010 for CFI. Under a strict interpretation, therefore, scalar invariance across activity levels, gender, and age is not given.

**Table 5:** Measurement invariance across activity levels, gender, and age.

**Models**	**MLR-χ^2^**	***df***	**CFI**	**SRMR**	**RMSEA [90% CI]**	**ΔCFI**	**ΔRMSEA**
**SAMPLES B AND C**							
Sample B (*n* = 788) (independent ESEM)	324.171	144	0.982	0.014	0.040 [0.034–0.046]	–	–
Sample C (*n* = 830) (independent ESEM)	330.155	144	0.984	0.012	0.040 [0.034–0.045]	–	–
Configural invariance (equivalence of factor structure)	649.746	288	0.983	0.013	0.039 [0.035–0.044]	–	–
Metric invariance (equivalence of factor loadings)	815.854	432	0.982	0.021	0.033 [0.030–0.037]	0.001	0.006
Scalar invariance (equivalence of the means of manifest variables)	888.885	458	0.980	0.027	0.034 [0.031–0.038]	0.003	0.005
**ACTIVITY LEVELS**							
Inactive group (*n* = 387) (independent ESEM)	349.642	144	0.974	0.015	0.049 [0.043–0.056]	–	–
Active group (*n* = 1223) (independent ESEM)	324.063	144	0.986	0.012	0.035 [0.030–0.040]	–	–
Configural invariance (equivalence of factor structure)	641.815	288	0.983	0.013	0.039 [0.035–0.043]	–	–
Metric invariance (equivalence of factor loadings)	762.153	432	0.984	0.021	0.031 [0.027–0.034]	−0.001	0.008
Scalar invariance (equivalence of the means of manifest variables)	1089.896	458	0.970	0.053	0.041 [0.038–0.045]	0.013	−0.002
**GENDER**							
Female group (*n* = 956) (independent ESEM)	319.807	144	0.986	0.012	0.036 [0.030–0.041]	–	–
Male group (*n* = 653) (independent ESEM)	295.248	144	0.982	0.014	0.040 [0.043–0.047]	–	–
Configural invariance (equivalence of factor structure)	614.562	288	0.985	0.013	0.038 [0.033–0.042]	–	–
Metric invariance (equivalence of factor loadings)	809.134	432	0.982	0.023	0.033 [0.029–0.036]	0.003	0.005
Scalar invariance (equivalence of the means of manifest variables)	1239.916	458	0.963	0.054	0.046 [0.043–0.049]	0.022	−0.008
**AGE**							
Adolescent group (*n* = 969)^a^ (independent ESEM)	399.006	144	0.981	0.013	0.043 [0.038–0.048]	–	–
Adult group (*n* = 633) (independent ESEM)	238.544	144	0.989	0.011	0.032 [0.025–0.039]	–	–
Configural invariance (equivalence of factor structure)	635.103	288	0.984	0.013	0.039 [0.035–0.043]	–	–
Metric invariance (equivalence of factor loadings)	833.799	432	0.981	0.024	0.034 [0.031–0.038]	0.003	0.005
Scalar invariance (equivalence of the means of manifest variables)	1142.825	458	0.968	0.046	0.043 [0.040–0.046]	0.016	−0.004

*All analyses are based on sample B (N = 788) and sample C (N = 830); MLR, robust maximum likelihood estimation; CFI, comparative fit index; SRMR, standardized root mean square residual; RMSEA, root mean square error of approximation; 90% CI = 90% confidence interval for RMSEA. ^a^Due to estimation problems, the residual variance of item aes2 was set >0*.

In conclusion, equal factor loadings and equal item loadings of the BMZI for adolescence and young adulthood across activity levels, gender, and age may be assumed. Therefore, the inventory can be applied to compare correlations of motives and goals between these groups; for instance, between women and men. Nevertheless, the item intercepts of the BMZI for adolescence and young adulthood differ between inactive and active people, females and males, and adolescents and young adults. These results are not surprising as they are in accordance with previous empirical studies showing mean differences across gender and age (Frederick-Recascino and Ryan, 1993; Campbell et al., 2001; Trujillo et al., 2004; Quindry et al., 2011; Stults-Kolehmainen et al., 2013; Molanorouzi et al., 2015). Overall, a clear interpretation of the mean differences in motives and goals of the BMZI for adolescence and young adulthood is difficult because it remains unclear to what extent these differences are caused by measurement-related differences or content-related facts.

## General Discussion

With three different samples constituting an overall total of 2,318 subjects, the BMZI for adolescence and young adulthood was adapted and psychometrically examined. Based on an expert focus group and a review of the literature relevant motives and goals were identified and potential items were developed. After the compilation of an initial item pool, the first ESEM analyses focused on the factorial structure of sample A and the adaptation of the item pool. As a consequence, the item pool was supplemented with three new items and checked again with respect to its factorial structure with sample B. The resulting eight-factor structure showed an excellent fit of the data and was successfully cross-validated in sample C. The final BMZI for adolescence and young adulthood contains 26 items covering eight motives and goals: Contact, Competition/Performance, Distraction/Catharsis, Body/Appearance, Fitness, Health, Aesthetics, and Risk/Challenge. The results also supported the reliability and discriminant validity of the BMZI for adolescence and young adulthood. Furthermore, the correlations between the BMZI factors and exercise- and sport-related self-concordance provided satisfactory evidence for the criterion validity of the BMZI for adolescence and young adulthood. Finally, the metric invariance of the questionnaire across activity levels, gender, and age was confirmed. In summary, the BMZI for adolescence and young adulthood may be considered a reliable and valid instrument to assess exercise- and sport-related motives and goals.

In comparison with the original BMZI for middle adulthood, the BMZI for adolescence and young adulthood has an additional motive and goal factor (Risk/Challenge) and differentiates the factors Fitness and Health. The targeted factor Activation/Enjoyment, however, did not emerge. An explanation for this phenomenon can be found in Self-Determination Theory proposed by Ryan and Deci (2017). They argue that the *What* (i.e., the specific content of people's goals) should be separated from the *Why*, the behavioral regulation of goal pursuits. For example, Contact or Competition/Performance may be seen as goal content, whereas Enjoyment can be interpreted as a behavioral regulation of goal pursuits. Based on this theoretical differentiation, the BMZI can also be linked to self-determination theory.

Particularly worthy of mention is the finding that motives and goals of the BMZI have the equal conceptual meaning for both adolescents and young adults (metric measurement invariance). This is somewhat surprising as there are numerous empirical findings and theoretical considerations proposing that adolescents and young adults differ with respect to biological, cognitive, and psychosocial aspects (Newman and Newman, 2012; Arnett, 2016). One explanation for this measurement equivalence could be that the developmental tasks of these two age group increasingly merge into one another and “the exact point when adolescents become adults can no longer be clearly identified” (Hurrelmann and Quenzel, 2015). In sum, the inventory can be used readily both with adolescents as well as young adults and is thus an economic solution to assess motives and goals.

Moreover, it is equally applicable to active and inactive people, which is of special significance because inactive individuals are a very important target group in the field of exercise and sport (Booth et al., 2012). Thus far, research has merely advocated the utilization of the BMZI for inactive people (Lehnert et al., 2011; Schmid et al., 2014); however, the empirical justification for the use of BMZI in adolescents and young adults can now be provided.

### Limitations and Future Research Directions

Some limitations of our research warrant further discussion. First, the samples used in this study could not be selected randomly. As a consequence, the sample was slightly biased because it was primarily composed of students and white-collar employees, while those in lower-status occupations (e.g., blue-collar workers) were underrepresented. Further evaluation of the BMZI for adolescence and young adulthood for this occupational group is needed. A second limitation relates to the two different types of data collection: paper-pencil and online. For example, the students in school classes could ask questions in cases of ambiguity, whereas individuals who completed the online-version did not have this opportunity. Although research has indicated that results are generally equivalent across paper-pencil and online data collection (Weigold et al., 2013), future studies should clarify whether this consistency is observed when the BMZI is used in adolescents and young adults.

Even if further research is warranted, in its present form, the BMZI for adolescence and young adulthood is an age-specific and psychometrically sound inventory that may be used in practice as well as in research. With respect to the practical application in the fields of exercise and sport, the BMZI for adolescents and young adults may be an economically viable and suitable tool to identify individual differences in exercise- and sport-related motives and goals. This knowledge can be used within sport counseling or in the conceptualization of specific interventions among target groups to promote exercise and sport (Sudeck et al., 2011; Krauss et al., 2017). Future research should investigate how such interventions must be designed to satisfy motives and goals. Outstanding issues concern the choice of suitable exercise and sport activities and their implementation (e.g., Ekkekakis, 2009). The promising modularity of the three BMZIs for adolescence and young adulthood, middle adulthood (Lehnert et al., 2011; Schmid et al., 2018), as well as late adulthood (Schmid et al., 2014) should enable researchers to track the development of exercise- and sport-related motives and goals across the whole lifespan.

## Author Contributions

JulS and AC contributed to the conception and design of the study. VG and JulS acquired the data. VG, JulS, and JürS performed the data analysis and interpreted the data. VG wrote the first draft of the manuscript. All authors revised the manuscript critically for important intellectual content, and read and approved the submitted version.

### Conflict of Interest Statement

The authors declare that the research was conducted in the absence of any commercial or financial relationships that could be construed as a potential conflict of interest.
