# Supplementary material for: The Bernese Motive and Goal Inventory for Adolescence and Young Adulthood
Source: Front Psychol. 2019 Jan 24;9:2785. doi: 10.3389/fpsyg.2018.02785 (PMC6357923; doi:10.3389/fpsyg.2018.02785)
Supplement: Supplementary file 1 [file Table_1.docx]

**ESM 1: Comparison of existing sport and exercise motive and goal questionnaires**

**Table 1. Comparison of existing sport and exercise motive and goal questionnaires**

| Questionnaire attribute | | **Attitude toward Physical Activity-D**  (Steffgen et al., 2000) | **Participation Motivation Questionnaire**  (Gill et al., 1983) | **Personal Incentives for Exercise Questionnaire**  (Duda and Tappe, 1989) | **Revised Motivation for Physical Activity Measure**  (Ryan et al., 1997) | **Exercise Motivation Inventory 2 for adolescence**  (Ingledew and Sullivan, 2002) | **Physical Activity and Leisure Motivation Scale** (Molanorouzi et al., 2014) | **Physical Activity and Leisure Motivation Scale Youth**  (Kueh et al., 2018) | **Goal content for exercise questionnaire**  (Sebire et al., 2008) | **Sport-interessenstest**  (Schmid et al., 2017) | **Berner Motiv- und Zielinventar**  (Lehnert et al., 2011) |
| --- | --- | --- | --- | --- | --- | --- | --- | --- | --- | --- | --- |
| Targeted age group | | Adolescence | Adolescence | No information | No information | Adolescence | No information | Adolescence | No information | Adolescence | Middle adulthood |
| Evaluation sample | | *N* = 2477 (42 % ♀), *M* = 15 years | *N* = 1138 (37 % ♀), *M* = 8-18 J. | *N* = 876 (60 % ♀), any information about age | *N* = 155 (57 % ♀), *M*= 20 years | *N* = 180 (51 % ♀), *M* = 15 years | *N* = 502 (57 % ♀), *M* = 31 years | *N* = 783 (57 % ♀), *M* = 14.5 years | *N* = 666 (57 % ♀), *M* = 34 J. | *N* = 1243 (53 % ♀), *M* = 15 years | *N* = 603 (59 % ♀), *M* = 49 years |
| Language | | German | English | English | English | English | English | Malay | English | German | German |
| Numbers of items /numbers of factors | | 36/5 | 30/8 | 48/9 | 30/5 | 48/13 | 40/8 | 28/7 | 20/5 | 75/13 | 24/7 |
| Reliability | | .60 ≤ α ≤ .80 | .30 ≤ α ≤ .78 | .74 ≤ α ≤ .94 | .78 ≤ α ≤ .92 | .56 ≤ α ≤ .92 | .75 ≤ α ≤ .92 | .73 ≤ α ≤ .85 | .75 ≤ α ≤ .92 | .71 ≤ α ≤ .87 | .72 ≤ α ≤ .90 |
| Data analysis | | EFA | EFA | EFA | EFA | CFA | CFA | CFA | EFA, CFA | EFA, CFA | EFA, CFA |
| Theoretical background | | Kenyon`s Structure Model | Literature research, interviews | Literature research, interviews, Theory of personal investment | Literature research, plausibility considerations | Loose connection with SDT; plausibility considerations | SDT | SDT | SDT | Holland`s Theory of Interest | Gabler`s Motive Taxonomy |
| Motives and goals | Competition/Achievement | Risk Competition/ Achievement | Achievement, Social status, Mastery | Competition/ Achievement | Achievement/ Challenge | Challenge | Mastery, Competition/Ego | Mastery, Competition/Ego | Mastery | Competition, Aggressiveness, Risk, Intellect, Spontaneity | Competition/ Achievement |
|  | Enjoyment | — | Enjoyment | — | Interest/Enjoyment | Enjoyment | Enjoyment | Enjoyment | — | — | Activation/ Enjoyment |
|  | Recreation | Recreation/ satisfaction | Energy reduction | Mental health | — | Stress management, Revitalization | Stress management, Revitalization | Psychological condition | — | — | Distraction/ Catharsis |
|  | Health | Physical health/ Fitness | — | Physical health | Physical health/ Fitness | Avoidance of diseases, Positive health, Strength, Endurance, Agility | Physical health/ Fitness | Physical health/ Fitness | Physical health/ Fitness | Endurance, Speed, Strength, Coordination, Fitness  (Figure/Physical health) | Physical health/ Fitness |
|  | Fitness |  | Fitness | Flexibility/Agility |  |  |  |  |  |  |  |
|  | Figure/ Appearance | — | — | Weight regulation, appearance | Figure/ Appearance | Weight regulation, Appearance | Figure/ Appearance | Figure/ Appearance | Figure/ Appearance |  | Figure/ Appearance |
|  | Social aspects | Affiliation/  Social interaction | Team, Significant others, Friends | Affiliation, Social recognition | Affiliation/ Social interaction | Affiliation, Social recognition, Social pressure | Affiliation, Social recognition | Affiliation | Affiliation, Social recognition | Community | Contact  (Affiliation, Social recognition) |
|  | Aesthetics | — | — | — | — | — | — |  | — | Artistic expression | Aesthetics |

*Note.* EFA = exploratory factor analysis; CFA = confirmatory factor analysis; SDT = Self-Determination Theory.

Some goal and motive designations were slightly adapted to provide a better overview.

# References

Duda, J. L., and Tappe, M. K. (1989). The personal incentives for exercise questionnaire: Preliminary development. *Perceptual and Motor Skills* 68, 1122.

Gill, D. L., Gross, J. B., and Huddleston, S. (1983). Participation motivation in youth sport. *International Journal of Sport Psychology* 14, 1–4.

Ingledew, D. K., and Sullivan, G. (2002). Effects of body mass and body image on exercise motives in adolescence. *Psychology of Sport and Exercise* 3, 323–338.

Kueh, Y. C., Abdullah, N., Kuan, G., Morris, T., and Naing, N. N. (2018). Testing measurement and factor structure invariance of the physical activity and leisure motivation scale for youth across gender. *Frontiers in Psychology* 9, 397. doi: 10.3389/fpsyg.2018.01096.

Lehnert, K., Sudeck, G., and Conzelmann, A. (2011). BMZI – Berner Motiv- und Zielinventar im Freizeit- und Gesundheitssport [BMZI – Bernese motive and goal inventory in leisure and health sports]. *Diagnostica* 57, 146–159. doi: 10.1026/0012-1924/a000043.

Molanorouzi, K., Khoo, S., and Morris, T. (2014). Validating the Physical Activity and Leisure Motivation Scale (PALMS). *BMC Public Health* 14, 1–12.

Ryan, R. M., Frederick, C. M., Lepes, D., Rubio, N., and Sheldon, K. M. (1997). Intrinsic motivation and exercise adherence. *International Journal of Sport Psychology* 28, 335–354.

Schmid, J., Albertin, K., Toggweiler, S., Birrer, D., Zimprich, D., and Seiler, R. (2017). Entwicklung und Validierung eines Fragebogens zur Erfassung von Sportinteressen im Jugendalter (SPIT) [Development and validation of a measure of sports interests in adolescence (SPIT)]. *Zeitschrift für Sportpsychologie* 24, 140–154. doi: 10.1026/1612-5010/a000202.

Sebire, S. J., Standage, M., and Vansteenkiste, M. (2008). Development and validation of the goal content for exercise questionnaire. *Journal of Sport & Exercise Psychology* 30, 353–377.

Steffgen, G., Fröhling, R., and Schwenkmezger, P. (2000). Motive sportlicher Aktivität: Psychometrische Untersuchungen einer Kurzform der ATPA-D-Skalen [Motives of sports activities: Psychometric examination of a short version of the ATPA-D-Scales]. *Sportwissenschaft* 30, 408–421.
