# Supplementary material for: The Bernese Motive and Goal Inventory for Adolescence and Young Adulthood
Source: Front Psychol. 2019 Jan 24;9:2785. doi: 10.3389/fpsyg.2018.02785 (PMC6357923; doi:10.3389/fpsyg.2018.02785)
Supplement: Supplementary file 2 [file Table_2.docx]

**ESM 2: Data Analysis and Results of Comparison of Non-Nested Models**

**Supplementary Data Analysis**

In a series of models with Sample A 5 to 11 factors were estimated using ESEM (Asparouhov and Muthén, 2009) in Mplus (Muthén and Muthén, 1998-2017). To compare the different models, content- and statistical criteria were applied. In addition to the relative fit indices CFI, RMSEA, and SRMR, the Akaike Information Criterion (AIC; Akaike, 1987) was used to compare non-nested models. The AIC considers model fit as well as model complexity and parsimony, respectively (Brown, 2006). Generally, we favoured models with a relatively low AIC, indicating both a better model fit and a more parsimonious model. However, content-related criteria were at least as important as statistical criteria for deciding the optimal factor solution. In light of the aim of the study, the development of an age-specific questionnaire, and its application in practice, we especially valued a parsimonious (economical) and yet comprehensive and detailed model consisting of clearly distinguishable motives and goals. Following the same procedure, a series of models with 5 to 9 factors with Sample B was conducted using ESEM.

**Supplementary Results and Brief Discussion**

Among Sample A, we chose Model C with 7 factors (see Table 1 and ESM 3). Overall, all the models had very good fit indices. However, Models A and B with 5 and 6 factors, respectively, did not cover a broad variety of motives and goals and thus did not allow an appropriate differentiation of the motives and goals in practice, whereas Model D, E, F, and G do not have clearly distinguishable factors.

Among Sample B, we opted for Model D with 8 factors since this solution had clear distinguishable factors and excellent fit indices (see Table 2). Models A and B with 5 and 6 factors, respectively, did not cover a wide variety of motives and goals. In contrast, the scientific value of Model E seems limited since the factors Competition and Performance were not clearly distinguishable. Finally, since both Models C and D had excellent fit indices, we therefore chose the 8-factor solution based on content-related criteria.

In light of the importance of health in today's society (Warburton and Bredin, 2017; Penedo and Dahn, 2005), a differentiation of the motives and goals for Health and Fitness seems useful. However, in the absence of hard-and-fast statistical criteria for choosing between non-nested models, we gave preference to theoretical considerations and criteria that are, of course, arguable.

**Table 1. Fit Indices and Exclusion Criteria of Competing Models**

| Model | AIC | CFI | RMSEA [90% CI] | SRMR | Factors | Exclusion criteria |
| --- | --- | --- | --- | --- | --- | --- |
| Original BMZI  (7 factors) | 45491.963 | .982 | .041 [.034–.047] | .016 | Contact, Competition/Performance, Distraction/Catharsis, Body/Appearance, Fitness/Health, Activation/Enjoyment, Aesthetics | Several cross-loadings: rischa4, actenj1-3  No clear factor Activation/Enjoyment |
| Model A  (5 factors) | 34113.301 | .980 | .049 [.041–.058] | .018 | Contact, Competition/Performance, Distraction/Catharsis, Body/Appearance, Fitness/Health | Low degree of differentiation: variety of sport-related motives and goals is not completely covered |
| Model B  (6 factors) | 38080.202 | .979 | .049 [.041–.057] | .016 | Contact, Competition/Performance, Distraction/Catharsis, Body/Appearance, Fitness/Health, Aesthetics | Low degree of differentiation: variety of sport-related motives and goals is not completely covered |
| Model C  (7 factors) | 42791.017 | .982 | .044 [.037–.051] | .016 | Contact, Competition/Performance, Distraction/Catharsis, Body/Appearance, Fitness, Health, Aesthetics | – |
| Model D  (8 factors) | 48161.423 | .983 | .040 [.034–.046] | .015 | Contact, Competition/Performance, Distraction/Catharsis, Body/Appearance, Fitness, Health, Aesthetics, Risk/Challenge | Cross-loadings: rischa4 |
| Model E  (9 factors) | 49795.207 | .987 | .036 [.029–.042] | .012 | Contact, Competition, Performance, Distraction/Catharsis, Body/Appearance, Fitness, Health, Aesthetics, Risk/Challenge | No clear distinguishable factors Competition, Performance as well as Risk/Challenge |
| Model F  (10 factors) | 52792.649 | .987 | .036 [.030–.042] | .011 | Contact, Competition, Performance, Distraction/Catharsis, Body Weight, Appearance, Fitness, Health, Aesthetics, Risk/Challenge | No clear distinguishable factors Body Weight as well as Appearance |
| Model G  (11 factors) | 56397.088 | .988 | .034 [.028–.040] | .010 | Contact, Competition, Performance, Distraction/Catharsis, Body Weight, Appearance, Fitness, Health, Activation/Enjoyment, Aesthetics, Risk/Challenge | No clear factor Activation/Enjoyment |

*Note. n* = 700 (Sample A).

**Table 2. Fit Indices and Exclusion Criteria of Competing Models**

| Model | AIC | CFI | RMSEA [90% CI] | SRMR | Factors | Exclusion criteria |
| --- | --- | --- | --- | --- | --- | --- |
| Model A  (5 factors) | 38917.508 | .975 | .054 [.047–.062] | .018 | Contact, Competition/Performance, Distraction/Catharsis, Body/Appearance, Fitness/Health | Low degree of differentiation: variety of sport-related motives and goals is not completely covered |
| Model B  (6 factors) | 43507.776 | .978 | .049 [.042–.057] | .016 | Contact, Competition/Performance, Distraction/Catharsis, Body/Appearance, Fitness/Health, Aesthetics | Low degree of differentiation: variety of sport-related motives and goals is not completely covered |
| Model C  (7 factors) | 49433.773 | .980 | .044 [.038–.051] | .015 | Contact, Competition/Performance, Distraction/Catharsis, Body/Appearance, Fitness/Health, Aesthetics, Risk/Challenge | No differentiation of Fitness and Health |
| Model D  (8 factors) | 54689.480 | .983 | .040 [.034–.045] | .014 | Contact, Competition/Performance, Distraction/Catharsis, Body/Appearance, Fitness, Health, Aesthetics, Risk/Challenge | — |
| Model E (9 factors) | 58462.078 | .984 | .039 [.033–.044] | .013 | Contact, Competition, Performance, Distraction/Catharsis, Body/Appearance, Fitness, Health, Aesthetics, Risk/Challenge | No clear distinguishable factors Competition and Performance |

*Note. n* = 788 (Sample B).

References

Akaike, H. (1987). Factor analysis and AIC. *Psychometrika* 52, 317–332.

Asparouhov, T., and Muthén, B. (2009). Exploratory structural equation modeling. *Structural Equation Modeling: A Multidisciplinary Journal* 16, 397–438. doi: 10.1080/10705510903008204.

Brown, T. A. (2006). *Confirmatory factor analysis for applied research.* New York, NY: Guilford Press.

Muthén, L. K., and Muthén, B. O. (1998-2017). “Mplus user`s guide,”. 8th ed.

Penedo, F. J., and Dahn, J. R. (2005). Exercise and well-being: A review of mental and physical health benefits associated with physical activity. *Current Opinion in Psychiatry* 18, 189–193. doi: 10.1097/00001504-200503000-00013.

Warburton, D. E. R., and Bredin, S. S. D. (2017). Health benefits of physical activity: A systematic review of current systematic reviews. *Current Opinion in Cardiology* 32, 541–556. doi: 10.1097/HCO.0000000000000437.
