# Supplementary material for: The Bernese Motive and Goal Inventory for Adolescence and Young Adulthood
Source: Front Psychol. 2019 Jan 24;9:2785. doi: 10.3389/fpsyg.2018.02785 (PMC6357923; doi:10.3389/fpsyg.2018.02785)
Supplement: Supplementary file 3 [file Table_3.docx]

**ESM 3: Standardized Factor Loadings of the Initial Factor Validity and Distribution Characteristics**

**Table 1. Standardized Factor Loadings of the Initial Factor Validity and Distribution Characteristics**

| Factor name | Items | Factors | | | | | | |  | | SMC | CR (ρ) | *M* | *SD* |
| --- | --- | --- | --- | --- | --- | --- | --- | --- | --- | --- | --- | --- | --- | --- |
|  |  | 1 | 2 | 3 | 4 | 5 | 6 | 7 | |  |  |  |  |  |
| Contact | con1: To be social with others. | .82 |  |  |  |  |  |  | |  | .73 | .90 | 3.28 | 1.28 |
|  | con2: To do something in a group. | .81 |  |  |  |  |  |  | |  | .75 |  | 3.02 | 1.34 |
|  | con3: To meet friends and acquaintances. | .83 |  |  |  |  |  |  | |  | .75 |  | 3.08 | 1.33 |
|  | con4: To get to know people.^a^ | .63 |  |  |  |  |  |  | |  | .52 |  | 2.43 | 1.18 |
|  | con5: To make new friends through exercise.^a^ | .56 |  |  |  |  |  |  | |  | .45 |  | 2.48 | 1.16 |
| Competition/ Performance | comper1: To compete with others. |  | .77 |  |  |  |  |  | |  | .62 | .78 | 2.29 | 1.25 |
|  | comper2: Because I thrive on competition. |  | .78 |  |  |  |  |  | |  | .68 |  | 2.37 | 1.32 |
|  | comper3: To achieve my exercise goals. |  | .60 |  |  |  |  |  | |  | .47 |  | 3.37 | 1.30 |
| Distraction/ Catharsis | discat1: To reduce anger and tension. |  |  | .71 |  |  |  |  | |  | .51 | .83 | 2.96 | 1.22 |
|  | discat2: To distract myself from other problems. |  |  | .57 |  |  |  |  | |  | .42 |  | 2.95 | 1.29 |
|  | discat3: To reduce stress. |  |  | .88 |  |  |  |  | |  | .81 |  | 3.49 | 1.16 |
|  | discat4: To organize my thoughts. |  |  | .72 |  |  |  |  | |  | .63 |  | 3.24 | 1.24 |
| Body/ Appearance | bodapp1: To lose weight. |  |  |  | .87 |  |  |  | |  | .79 | .88 | 2.54 | 1.35 |
|  | bodapp2: To regulate my weight. |  |  |  | .86 |  |  |  | |  | .81 |  | 2.70 | 1.37 |
|  | bodapp3: Because of my body shape. |  |  |  | .68 | .25 |  |  | |  | .64 |  | 2.97 | 1.30 |
| Health | hea1: Primarily for health reasons. |  |  |  |  |  | .69 |  | |  | .66 | .80 | 3.36 | 1.14 |
|  | hea2: Primarily to improve my state of health. |  |  |  |  |  | .88 |  | |  | .88 |  | 3.38 | 1.17 |
|  | hea3: To work against physical health problems. |  |  |  |  |  | .44 |  | |  | .35 |  | 3.11 | 1.21 |
| Fitness | fit1: To keep myself in good physical shape. |  |  |  |  | .67 |  |  | |  | .59 | .86 | 4.34 | 0.86 |
|  | fit2: Primarily to be fit. |  |  |  |  | .74 |  |  | |  | .70 |  | 4.10 | 0.92 |
|  | fit3: Primarily to do something for my physical fitness. |  |  |  |  | .72 | .20 |  | |  | .76 |  | 4.08 | 0.92 |
| Aesthetics | aes1: Because exercise offers the possibility for beautiful movements. |  |  |  |  |  |  | .91 | |  | .82 | .84 | 2.23 | 1.19 |
|  | aes2: To experience beautiful movements. |  |  |  |  |  |  | .79 | |  | .67 |  | 2.62 | 1.30 |

*Notes.* *N =* 700 (Sample A). Factor 1: Contact; Factor 2: Competition/Performance; Factor 3: Distraction/Catharsis; Factor 4: Body/Appearance; Factor 5: Fitness; Factor 6: Health; Factor 7: Aesthetics; SMC = squared multiple correlation; CR = composite reliability. Factor loadings < .20 are not presented.

^a^In accordance with the studies of the BMZI for middle adulthood (Lehnert et al., 2011; Schmid et al., 2018) and of the BMZI for older adulthood (Schmid et al., 2014), we admitted an error covariance between con4 and con5.

Scale ranging from 1 to 5.
