# Supplementary material for: The Bernese Motive and Goal Inventory for Adolescence and Young Adulthood
Source: Front Psychol. 2019 Jan 24;9:2785. doi: 10.3389/fpsyg.2018.02785 (PMC6357923; doi:10.3389/fpsyg.2018.02785)
Supplement: Supplementary file 4 [file Data_Sheet_1.docx]

**Appendix: Initial Item Inventory of the BMZI for Adolescence and Young Adulthood**

**Table 1. Initial Item Inventory of the BMZI for Adolescence and Young Adulthood**

| Intended motives and goals | Item | German version | English version | Source |
| --- | --- | --- | --- | --- |
| Contact | con1^a^ | Um mit anderen gesellig zusammen zu sein. | To be social with others. | Lehnert et al. (2011) |
|  | con2^a^ | Um etwas in einer Gruppe zu unternehmen. | To do something in a group. | Lehnert et al. (2011) |
|  | con3^a^ | Um dabei Freunde/Bekannte zu treffen. | To meet friends and acquaintances. | Lehnert et al. (2011) |
|  | con4^a^ | Um dadurch Menschen kennen zu lernen. | To get to know people. | Lehnert et al. (2011) |
|  | con5^a^ | Um durch den Sport neue Freunde zu gewinnen. | To make new friends through exercise. | Lehnert et al. (2011) |
| Competition/ Performance | comper1^a^ | Um mich mit anderen zu messen. | To compete with others. | Lehnert et al. (2011) |
|  | comper2^a^ | Weil ich im Wettkampf aufblühe. | Because I thrive on competition. | Lehnert et al. (2011) |
|  | comper3^a^ | Um sportliche Ziele zu erreichen. | To achieve my exercise goals. | Lehnert et al. (2011) |
|  | comper4 | Um bessere Leistungen als andere zu erreichen. | To achieve better performances than others. | Rogers (2000) |
|  | comper5 | Um meine sportlichen Fähigkeiten zu verbessern | To improve my physical skills. | Gill et al. (1983) |
|  | comper6 | Um meine Technik in einer Sportarten zu verbessern. | To improve my technique in a sporting activity. | Gill et al. (1983) |
|  | comper7^c^ | Um mein Leistungsniveau zu erhöhen. | To increase my level of performance. | Own formulation |
| Distraction/ Catharsis | discat1^a^ | Um Ärger und Gereiztheit abzubauen. | To reduce anger and tension. | Lehnert et al. (2011) |
|  | discat2^a^ | Weil ich mich so von anderen Problemen ablenke. | To distract myself from other problems. | Lehnert et al. (2011) |
|  | discat3^a^ | Um Stress abzubauen. | To reduce stress. | Lehnert et al. (2011) |
|  | discat4^a^ | Um meine Gedanken im Kopf zu ordnen. | To organize my thoughts. | Lehnert et al. (2011) |
| Body/ Appearance | bodapp1^a^ | Um abzunehmen. | To lose weight. | Lehnert et al. (2011) |
|  | bodapp2^a^ | Um mein Gewicht zu regulieren. | To regulate my weight. | Lehnert et al. (2011) |
|  | bodapp3^a^ | Wegen meiner Figur. | Because of my body shape. | Lehnert et al. (2011) |
|  | bodapp4^b^ | Um einen attraktiven Körper zu haben. | To have an attractive body. | Cash and Labarge (1996) |
|  | bodapp5^b^ | Um mein körperliches Aussehen zu verbessern | To improve my physical appearance. | Sebire et al. (2008) |
|  | bodapp6^b^ | Um einen sportlichen Körper zu haben. | To have an athletic body. | Own formulation |
|  | bodapp7^b^ | Um meinen Körper zu formen. | To shape my body. | Schmid et al. (2017) |
| Health | hea1^a^ | Vor allem um meinen Gesundheitszustand zu verbessern. | Primarily for health reasons. | Lehnert et al. (2011) |
|  | hea2^a^ | Vor allem aus gesundheitlichen Gründen. | Primarily to improve my state of health. | Brehm and Pahmeier (1998) |
|  | hea3^a^ | Um körperlichen Beschwerden entgegenzuwirken. | To work against physical health problems. | Own formulation |
| Fitness | fit1^a^ | Um mich in körperlich guter Verfassung zu halten. | To keep myself in good physical shape. | Lehnert et al. (2011) |
|  | fit2^a^ | Vor allem um fit zu sein. | Primarily to be fit. | Lehnert et al. (2011) |
|  | fit3^a^ | Vor allem um etwas für meine körperliche Fitness zu tun. | Primarily to do something for my physical fitness. | Brehm and Pahmeier (1998) |
| Activation/ Enjoyment | actenj1^b^ | Um mich zu entspannen. | To relax me. | Lehnert et al. (2011) |
|  | actenj2^b^ | Vor allem aus Freude an der Bewegung. | Primarily due to joy of move. | Lehnert et al. (2011) |
|  | actenj3^b^ | Um neue Energie zu tanken. | To replenish new energy. | Lehnert et al. (2011) |
| Aesthetics | aes1^a^ | Weil Sport mir die Möglichkeit für schöne Bewegungen bietet. | Because exercise offers the possibility for beautiful movements. | Lehnert et al. (2011) |
|  | aes2^a^ | Um schöne Bewegungen zu erleben. | To experience beautiful movements. | Own formulation |
|  | aes3 | Um harmonische Bewegungen zu erleben. | To experience harmonious movements. | Own formulation |
| Risk/ Challenge | rischa1^ac^ | Um etwas zu wagen. | To dare to do something. | Zaleskiewicz (2001) |
|  | rischa2^a^ | Weil riskante Situationen für mich reizvoll sind. | Because risky situations appeal to me. | Own formulation |
|  | rischa3^a^ | Um meinen Mut zu testen. | To test my courage. | Lehnert et al. (2011) |
|  | rischa4 | Wegen des Nervenkitzels. | For the thrill. | Lehnert et al. (2011) |
|  | rischa5^c^ | Weil mich gefährliche Situationen reizen. | Because dangerous situations appeal to me. | Schmid et al. (2017) |

^a^ Items of the final BMZI for adolescence and young adulthood. ^b^ Item was removed after the examination of the initial factor validity. ^c^ Item was added after the examination of the initial factor validity.

**References for the initial item inventory**

Brehm, W., and Pahmeier, I. (1998). “Sinnzuschreibungen, Konsequenz- und Kompetenzerwartungen,” [Attribution of meanings, consistency and competence expectations] in *Gesundheitssport: Ein Handbuch* [Health sports: A handbook], ed. K. Bös, W. Brehm, and D. Alfermann (Schorndorf: Hofmann), 221–230.

Cash, T. F., and Labarge, A. S. (1996). Development of the appearance schemas inventory: A new cognitive body-image assessment. *Cognitive Therapy and Research* 20, 37–50.

Gill, D. L., Gross, J. B., and Huddleston, S. (1983). Participation motivation in youth sport. *International Journal of Sport Psychology* 14, 1–4.

Lehnert, K., Sudeck, G., and Conzelmann, A. (2011). BMZI – Berner Motiv- und Zielinventar im Freizeit- und Gesundheitssport [BMZI – Bernese motive and goal inventory in leisure and health sports]. *Diagnostica* 57, 146–159. doi: 10.1026/0012-1924/a000043.

Rogers, H. E. (2000). “Development of recreational exercise motivation questionnaire (PhD thesis)”, School of Human Movement, Recreation, and Performance, Victoria University of Technology.

Schmid, J., Albertin, K., Toggweiler, S., Birrer, D., Zimprich, D., and Seiler, R. (2017). *Entwicklung und Validierung eines Fragebogens zur Erfassung von Sportinteressen im Jugendalter* (SPIT) [Development and validation of a measure of sports interests in adolescence (SPIT)]. *Zeitschrift für Sportpsychologie* 24, 140–154. doi: 10.1026/1612-5010/a000202.

Sebire, S. J., Standage, M., and Vansteenkiste, M. (2008). Development and validation of the goal content for exercise questionnaire. *Journal of Sport & Exercise Psychology* 30, 353–377.

Zaleskiewicz, T. (2001). Beyond risk seeking and risk aversion: Personality and the dual nature of economic risk taking. *European Journal of Personality* 15, 105–122.
